# Butylated Hydroxytoluene (BHT) and p-Coumaric Acid Conjugates of Dipeptide Proline and GABA as Multi-Functional Agents with High Pharmacological Potential

**DOI:** 10.3390/molecules31081323

**Published:** 2026-04-17

**Authors:** Georgios Papagiouvannis, Panagiotis Theodosis-Nobelos, Eleni A. Rekka

**Affiliations:** 1 Department of Pharmacy, School of Health Sciences, Frederick University, Nicosia 1036, Cyprus; hsc.np@frederick.ac.cy; 2 Department of Pharmaceutical Chemistry, School of Pharmacy, Aristotle University of Thessaloniki, 54124 Thessaloniki, Greece; rekka@pharm.auth.gr

**Keywords:** oxidative stress, inflammation, multi-targeting compounds, proline, GABA, p-coumaric acid, BHT

## Abstract

Oxidative stress and inflammation are interconnected pathological processes involved in the progression of neurodegenerative, cardiovascular, and metabolic diseases, highlighting the need for multifunctional therapeutic agents targeting multiple pathways. In this study, two novel hybrid compounds were designed and synthesized in three steps by conjugating butylated phenolic moieties derived from butylated hydroxytoluene and p-coumaric acid with proline and γ-aminobutyric acid (GABA). The aim was the combination of antioxidant, anti-inflammatory, and cytoprotective properties within a single molecular framework. The compounds were evaluated using a comprehensive panel of in vitro and in vivo assays to assess antioxidant, metal-reducing, iron-chelating, antiglycation, anti-inflammatory, and acetylcholinesterase inhibitory activities. Both compounds exhibited significant antioxidant activity, with compound **2** demonstrating superior radical scavenging ability against DPPH, ABTS·^+^ and hydrogen peroxide (IC_50_ 86 μM, 25 μM and 104 μM, respectively), enhanced ferric-reducing capacity (up to 91% of trolox activity), and strong iron-chelating activity (61.3%). Compound **2** also showed potent inhibition of lipid peroxidation (IC_50_ 17.5 μM) and moderate antiglycation effects (44%), indicating substantial cytoprotective potential. Furthermore, both compounds selectively inhibited COX-2 over COX-1 and demonstrated moderate lipoxygenase inhibition, while compound **2** exhibited significant in vivo anti-inflammatory activity (53%), exceeding that of ibuprofen. Moderate acetylcholinesterase inhibition was also observed. In summary, the results confirm the design rationale, indicating that compound **2** could be further optimized as a multi-targeting molecule directed against oxidative stress- and inflammation-mediated conditions.

## 1. Introduction

Oxidative stress and inflammation are interrelated processes implicated in the pathogenesis of various chronic diseases. Oxidative stress derives from an imbalance between reactive oxygen species (ROS) production and antioxidant defense, leading to cellular damage [1]. This oxidative damage can activate signaling pathways, such as Nuclear Factor-κΒ (NF-κB), which upregulate pro-inflammatory cytokines [2]. Conversely, chronic inflammation can enhance ROS generation by activated immune cells, such as macrophages, increasing oxidative stress [3,4]. This bidirectional relationship creates a self-amplifying cycle contributing to tissue injury and disease progression, including cardiovascular disease, neurodegeneration, and cancer [5].

Oxidative stress and inflammation are main contributors to the development of cardiovascular diseases and diabetes. In diabetes, chronic hyperglycemia elevates ROS production, leading to endothelial dysfunction and vascular damage [6]. This oxidative stress activates pro-inflammatory pathways, resulting in the release of cytokines which exacerbate insulin resistance and promote atherosclerosis, whilst lipids deterioration and deranged production also add towards endothelial lesions [7,8]. In the cardiovascular system, excessive ROS impairs nitric oxide production and leads to its oxidation (production of the toxic peroxynitrite), promoting vasoconstriction and hypertension [9]. Moreover, oxidative stress-induced activation of the nucleotide-binding domain, leucine-rich-containing family, pyrin domain-containing-3 (NLRP3) inflammasome contributes to inflammation and fibrosis, key features of inflammatory conditions like diabetic cardiomyopathy. Additionally, the role of ferroptosis is highlighted, an iron-dependent form of cell death driven by lipid peroxidation, in diabetic cardiac microvascular dysfunction [10]. Targeting oxidative stress and inflammation through lifestyle interventions and pharmacological agents, such as sodium/glucose cotransporter 2 (SGLT2) inhibitors, have shown promise in mitigating cardiovascular complications which may be associated with metabolic conditions like diabetes [11].

Neurodegenerative disorders refer to a group of diseases marked by the progressive loss and malfunction of neurons. As global life expectancy rises, the incidence of such disorders also increases, highlighting the need for more effective therapeutic strategies [12]. Among these conditions, Alzheimer’s Disease (AD) stands out as the most prevalent, representing roughly 70% of dementia cases worldwide [13]. Oxidative stress significantly contributes to neurodegeneration, especially in the brain, which consumes over 20% of the inhaled oxygen [14]. Accumulated oxidative damage and mitochondrial impairment are key elements in AD progression, alongside with Aβ aggregation and tau hyperphosphorylation [15,16]. Additionally, overstimulation of N-methyl-D-aspartate (NMDA) receptors and excitotoxicity intensify oxidative stress, promoting neuronal damage [17]. Neuroinflammation, driven by microglia and astrocyte activation, also fosters neural loss [18], while prostaglandins and lipoxygenase activity gives rise to amyloid and tangle formation [19,20]. The cholinergic hypothesis links reduced acetylcholine to cognitive decline [21], and though acetylcholinesterase AchE inhibitors offer symptom relief [22], they fall short of providing a cure, necessitating further research into their role in AD, and the need for new drug treatment apart from symptomatic relief options [23].

Cyclooxygenases (COX-1 and COX-2) and lipoxygenases (particularly 5-LOX) are pivotal in the biosynthesis of pro-inflammatory eicosanoids, such as prostaglandins and leukotrienes, which are implicated in the pathogenesis of cardiovascular and neurodegenerative diseases [24,25]. Additionally, in neurodegenerative diseases like Alzheimer’s and Parkinson’s, overexpression of COX-2 and 5-LOX leads to increased production of neurotoxic mediators, deteriorating the neuronal damage [24]. Concerning the cardiovascular system, elevated 5-LOX expression has been observed in advanced atherosclerotic lesions, contributing to vascular inflammation and plaque instability, whilst 5-LOX inhibitors seem to attenuate the inflammatory and apoptotic marker expression, with improvement in the cognitive function and reduced tau pathology [26]. Similarly, COX-2 inhibitors have demonstrated neuroprotective effects by attenuating neuroinflammation and preserving blood–brain barrier integrity at post-ischemic tissues [27]. These findings underscore the therapeutic potential of targeting COX and LOX pathways to mitigate inflammation-driven damage in cardiovascular and neurodegenerative disorders. However, since COX inhibition may lead to the metabolism of arachidonic acid via the LOXs pathways, it has been deduced that dual COX and LOX inhibitory potency of the compounds used for degenerative conditions (central and peripheral cases) may add to the synergistic activity [28,29].

Butylated hydroxytoluene (BHT) is a synthetic phenolic compound widely recognized for its potent antioxidant properties. It functions by donating hydrogen atoms to neutralize free radicals, thereby terminating lipid peroxidation chain reactions and stabilizing cell membranes [30]. Recent studies have highlighted BHT’s protective role against ferroptosis, a form of regulated cell death associated with oxidative stress, inhibiting lipid peroxidation in neuronal cells [31]. In vivo experiments further demonstrate BHT’s neuroprotective effects in AD models, where it mitigates oxidative damage and modulates the expression of apoptosis related genes [32]. Additionally, BHT moiety and its derived conjugates exhibit anti-inflammatory activity by inhibiting enzymes like acetylcholinesterase, lipoxygenase and monoamine oxidase, which are implicated in neurodegenerative disorders [33,34].

p-Coumaric acid (p-CA), a phenolic compound prevalent in fruits, vegetables, and cereals, exhibits significant antioxidant, anti-inflammatory and cellular protective properties [35,36]. It effectively scavenges ROS and chelates metal ions, thereby mitigating oxidative stress [35]. In vitro studies have demonstrated that p-CA enhances the activity of antioxidant enzymes such as superoxide dismutase (SOD) and catalase in neuronal cells, offering protection against corticosterone-induced neurotoxicity [35]. Additionally, p-CA has been shown to reduce levels of pro-inflammatory cytokines like tumor necrosis factor-α (TNF-α) and interleukin-1β (IL-1β) in various models, including renal ischemia–reperfusion injury [37]. These findings underscore p-CA’s potential as a therapeutic agent in managing oxidative stress and inflammation-related conditions.

L-Proline is an amino acid which prevents the inflammatory effects of lipopolysaccharide (LPS) in the cerebral cortex and the cerebellum. It may also offer protection against ROS and reactive nitrogen species (RNS) and apoptosis, preventing calcium influx in the cytoplasm, endonucleases release and cytoskeleton degradation [38,39]. Additionally, proline conjugates seem to confer increase in the antioxidants, anti-inflammatory and hypolipidemic properties of the parent molecules when L-proline is introduced [40,41,42]. Proline amides have shown cellular protection and inhibition of enzymes linked to diabetes and its manipulation [43]. Moreover, GABA, the main inhibitory neurotransmitter in the central nervous system (CNS) [15], and its derivatives can reduce nociceptive and inflammatory responses [44]. On the contrary, inhibition of GABAergic neurotransmission generates brain oxidative stress, which is important in the etiology of seizure-induced neuronal death [45]. Furthermore, treatment with GABA reduced kainic acid stress induced COX-2 expression in cells and significantly reduced COX-2 and prostaglandin E2 production [46].

**Figure 1:**
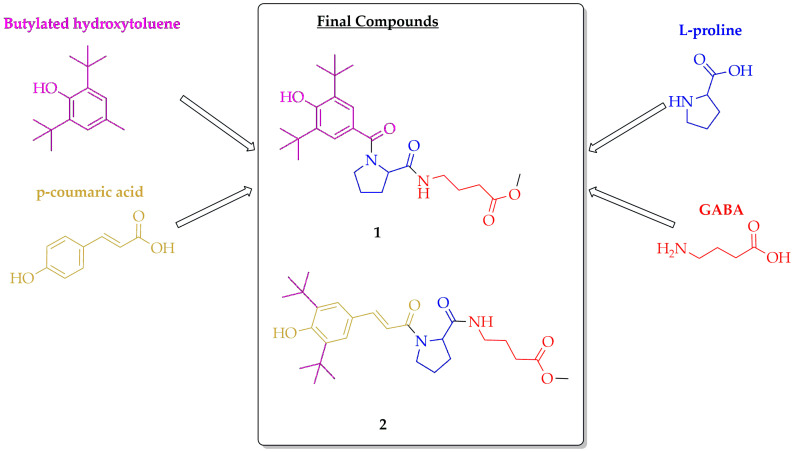
Depiction of the synthesized molecules and their parent molecules.

Taking into account the above-mentioned evidence and the multi-targeting compounds approach [47] we designed and synthesized two novel final compounds derived by amidation of proline and GABA with 3,5-di-tert-butyl-4-hydroxybenzoic acid (BHBA) and (*E*)-3-(3,5-di-*tert*-butyl-4-hydroxyphenyl)acrylic acid (BHCA). The former is a derivative of BHT (Figure 1) and the later of BHT and p-coumaric acid (Figure 1). Through the design of these molecules, we aim to incorporate three biologically active moieties (proline, GABA and the corresponding acid) into one entity, able to confer antioxidant, anti-inflammatory and cellular protective characteristics, that could be of great interest for the effective treatment of degenerative conditions, including neurodegenerative, cardiovascular and metabolic diseases and their derived derangements. Our approach aims to integrate complementary pharmacophores within a single molecular framework, thereby enhancing the potential for multi-target activity. The scientific validity of this approach is supported by the systematic synthesis and comprehensive biological evaluation of the resulting compounds, employing a broad panel of in vitro and in vivo assays. Such an integrated methodology enables a reliable assessment of their antioxidant, anti-inflammatory, and cytoprotective properties, providing a solid foundation for the exploration of these hybrids as candidates for complex disease management. Both compounds (Figure 1) were tested as antioxidants for their radical and reactive oxygen and nitrogen species scavenging potency [ability to interact with the stable free radicals 2,2-diphenyl-1-picrylhydrazyl (DPPH) and 2,2′-azinobis-(3-ethylbenzothiazoline-6-sulfonic acid (ABTS), and the hydrogen peroxide], together with their electron donating (ability to reduce ferric and molybdenum VI ions) and iron chelating capacity. Additionally, their ability to protect cellular components from oxidation or glycosylation in the presence of high levels of sugars, like the case of diabetes mellitus, is tested, as their inhibitory activity against rat hepatic microsomal membrane lipid peroxidation and their potency against fructose-induced protein glycation decrease. Moreover, they were examined for their inhibition against AchE, while their anti-inflammatory properties were evaluated by COX-1, COX-2 and LOX inhibition capacities and their in vivo effect on rat paw edema, induced by carrageenan.

## 2. Results and Discussion

### 2.1. Synthesis

**Scheme 1:**
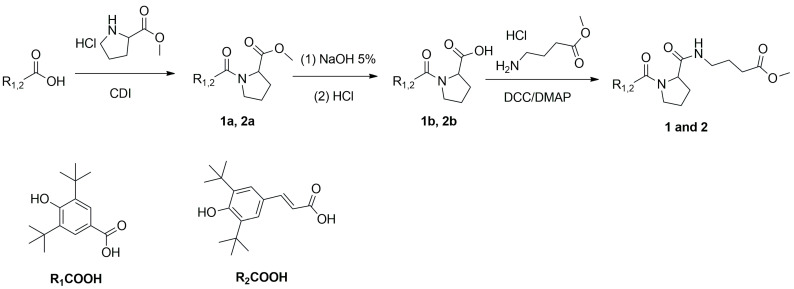
Synthetic procedure of compounds **1** and **2**.

The synthesis of the desired derivatives was carried out through a three-step process. In the first step, the appropriate carboxylic acid was coupled with methyl pyrrolidine-2-carboxylate hydrochloride (proline methyl ester) in the presence of carbonyldiimidazole (CDI) as the coupling agent, using dichloromethane as the reaction medium. The resulting intermediates were isolated in high yields via flash column chromatography. In the second step, ester intermediates **1a** and **2a** were subjected to hydrolysis with a 5% aqueous sodium hydroxide solution in 1,4-dioxane. After acidification, the resulting carboxylic acids were extracted with ethyl acetate, yielding over 90%. In the final step, compounds **1b** and **2b** were amidated with 4-aminobutanoate hydrochloride (GABA methyl ester) using dicyclohexylcarbodiimide (DCC) and dimethylaminopyridine (DMAP) in dichloromethane. The final products were purified by flash chromatography, with moderate yields, the highest reaching 78%. The synthetic procedure is illustrated in Scheme 1.

### 2.2. Radical Scavenging Assays

The synthesized molecules were evaluated for their reductive activity towards DPPH and ABTS·^+^ radicals, as well as for their hydrogen peroxide scavenging potency. Their IC_50_ values are shown in Table 1.

Taking IC_50_ values into account, compound **2** seems to have higher antioxidant potential in all three assays. Both compounds can reduce ABTS·^+^ radical and their efficacy is lower, but comparable, with trolox’s potency. The more extended conjugation effect and electron delocalization in compound **2** leads to a more stable phenolic radical. As a result, compound **2** possess higher antioxidant activity and reduces ABTS·^+^ more effectively. As far as DPPH assay is concerned, both molecules have also lower but comparable activity with trolox, but their IC_50_ values are relatively higher than those in the ABTS·^+^ assay. Although DPPH is a lipid soluble free radical and the tested compounds are expected to possess higher scavenging activity, due to their high lipophilicity (clogP 4.78 and 5.22 respectively), rigidity and steric hindrance in both DPPH and the compounds (*tert*-butyl groups are bulky substituents) may not allow the effective approach of the scavengers towards the free radical, restraining the electron transfer and therefore DPPH reduction. Finally, the antioxidant activity of the synthesized molecules was estimated by their ability to interact with H_2_O_2_. Hydrogen peroxide is produced normally through the activity of various enzymes, e.g., monoaminoxidases, and leads to the generation of more dangerous ROS, such as hydroxyl radical [48]. As expected, compound **2** could interact more effectively with hydrogen peroxide than compound **1**, due to its higher antioxidant capacity, exerting an IC_50_ value similar to that of ascorbic acid.

*tert*-Butyl substituents seem to enhance the antioxidant capacity, due to their hyperconjugation effect, which stabilizes the generated phenolic radical. Indeed, p-coumaric acid (4-hydroxycinnamic acid) was found not to interact so strongly with DPPH and H_2_O_2_ (40% and 30% respectively at 100 μΜ). In contrast, p-coumaric acid could reduce ABTS·^+^ radical as much as compound **2** (92% at 100 μΜ). In addition, the synthesized compounds, especially compound **2**, were more active than butylated hydroxytoluene (BHT) and butylated hydroxyanisole (BHA), whose antioxidant potential was similar to p-coumaric acid in all three assays, pointing out that amidation of butylated phenolic carboxylic acids can significantly elevate their antioxidant properties [49].

Moreover, cinnamic acid showed extremely low activity as free radical scavenger, in contrast to the phenolic cinnamic derivatives designed in our research. Finally, 4-hydroxybenzoic acid exerted lower activity towards both DPPH (IC_50_ could not be calculated) and ABTS·^+^ (IC_50_ 52.7 μM) than compound **1**, confirming the hypothesis that the *tert*-butyl groups enhance the antioxidant potency of the synthesized compounds [50].

### 2.3. Metal Cation Reducing Activity

To investigate the reducing capacity of the tested compounds toward ferric ions, two complementary experimental assays were performed. Specifically, the ferric-reducing antioxidant power (FRAP) assay was employed to assess the reduction in FeCl_3_, whereas the reducing power (RP) assay was used to evaluate the reduction in K_3_[Fe(CN)_6_]. Additionally, the total antioxidant capacity (TAC) assay was conducted in order to further examine their ability to reduce the Mo^6+^ cation. The activity of the compounds, expressed as percent activity of trolox, in all three assays is depicted in Table 2.

Compound **2** exhibited higher reducing activity than compound **1** across all applied assays, in agreement with the results obtained from the previously described ROS scavenging experiments. In contrast, compound **1** displayed a very limited ability to reduce ferric cations, both in the form of FeCl_3_ and ferricyanide. Notably, compound **2** was able to efficiently reduce ferric chloride in the FRAP assay, demonstrating reducing activity comparable to that of the reference antioxidant trolox, particularly at higher concentrations. However, its reducing efficacy in the RP assay was markedly lower, suggesting that compound **2** is more effective in reducing ferric salts than ferric ions complexed with cyanide ligands. Furthermore, a concentration-dependent decrease in reducing activity was observed for both assays, with diminished effects at lower concentrations, indicating the lack of intrinsic reducing activity and the inability of the molecule to maintain its reductive capacity upon dilution. In the TAC assay, compound **1** exhibited comparatively low reducing activity across the tested concentrations. In contrast, compound **2** demonstrated a pronounced reducing capacity, reducing phosphomolybdate cations (Mo^6+^) by up to 70% of the trolox standard, and importantly, maintaining substantial activity even at lower concentrations. This concentration-independent behavior suggests a strong intrinsic redox potential for compound **2**. Similar to how ferric cations function in the FRAP assay, molybdenum cations serve as salt in the TAC assay, confirming that compound **2** can reduce free cations rather than those in complexed forms.

### 2.4. Iron-Binding Ability

Iron chelation can significantly enhance the antioxidant potential of compounds by preventing the catalytic role of iron in ROS formation. Free iron, particularly Fe^2+^, participates in the Fenton reaction, which converts hydrogen peroxide into the highly reactive hydroxyl radicals capable of damaging lipids, proteins, and DNA. Chelating agents bind strongly to iron ions, reducing their availability and preventing their participation in redox cycling and radical generation [51]. As a result, iron binding indirectly decreases oxidative stress and inhibits iron-mediated lipid peroxidation and cellular damage. Additionally, iron chelators may enhance antioxidant defense by stabilizing metal ions and reducing ROS production, thereby protecting biological systems from oxidative injury. Therefore, compounds with iron-binding ability often exhibit improved antioxidant activity through both metal sequestration and suppression of radical-forming reactions [52]. Binding activity of the synthesized compounds as well as of the ethylenediaminetetraacetic acid (EDTA) is depicted in Table 3.

Compound **2** exhibited iron-binding activity exceeding fifty percent of that observed for EDTA and nearly twice the activity measured for compound **1**, indicating a significantly enhanced ability to interact with iron cations compared to the structurally related analog. One important structural feature that may contribute to this behavior is the presence of a phenolic hydroxyl group. Upon deprotonation, this functional group forms a phenolate anion, which possesses increased electron density and can effectively donate a lone pair of electrons to a metal center. In this way, the molecule can function as a monodentate ligand, coordinating to iron ions through the oxygen atom. The extent of deprotonation appears to be greater in compound **2**, likely due to improved stabilization of the resulting phenolate anion through electronic effects within the molecular framework. This enhanced stabilization increases the availability of electron density for metal coordination, thereby improving the iron-binding capability of compound **2** relative to compound **1**. However, due to steric hindrance, the interaction with the hydroxyl groups seems unfavorable. In addition to the phenolic group, the molecules contain two carbonyl functionalities derived from the starting carboxylic acid (benzyl- and phenylacryloyl- carbonyl groups respectively for compounds **1** and **2**) and proline moieties, both of which may serve as additional coordination sites for iron ions. Carbonyl oxygen atoms can act as Lewis bases, donating electron density to the metal center and contributing to complex formation. Notably, the carbonyl group within the phenylacryloyl moiety is involved in a more extensive conjugated system, which can increase electron delocalization and enhance the electron density on the oxygen atom. This increased electron density improves its ability to interact with iron cations. In that case, greater steric hindrance in compound **1** (tert-butyl substituents and the phenyl ring are closer to the carbonyl groups) could be an explanation for the lower binding potential of compound **1**. In addition, carbonyl groups derived from proline and GABA moieties could serve as coordination sites as well. Consequently, the combined effects of enhanced phenolate stabilization and the presence of electron-rich carbonyl groups, particularly the conjugated acryloyl carbonyl moiety, may provide a reasonable explanation for the superior activity observed for compound **2**.

### 2.5. Cellular Components Protective Potential

The protective potency of the designed molecules was evaluated using two experimental protocols. First, their inhibitory activity against lipid peroxidation in rat liver microsomal membranes was assessed. In addition, their ability to inhibit Cu^2+^-induced oxidative protein glycation was examined. The results are summarized in Table 4.

In accordance with all previous experiments, compound **2** exerted greater inhibitory activity against both lipid peroxidation and protein glycation. Inhibition of lipid peroxidation confers cytoprotecting activity by reducing oxidative damage to membrane phospholipids, preserving membrane integrity, and preventing regulated cell death pathways. Radical-trapping inhibitors of lipid peroxidation, including ferrostatin-1 and liproxstatin-1, markedly suppress lipid hydroperoxide accumulation and protect cells from oxidative stress-induced cytotoxicity in vitro. Thus, targeting lipid peroxidation represents a key antioxidant defense strategy to enhance cellular survival under conditions of oxidative challenge [53]. The high lipophilicity of both compounds allows their effective approach to lipid membranes, but the greater antioxidant potential of compound **2**, due to the most effective stabilization of the produced phenolic radical, could justify its higher inhibitory activity against lipid peroxidation.

Moreover, inhibition of oxidative protein glycation promotes cytoprotection by reducing the formation of advanced glycation end-products (AGEs) that otherwise induce oxidative stress, protein cross-linking, dysfunction, and inflammatory signaling. Glycation inhibitors, including antioxidants and reactive carbonyl scavengers, impede glycation pathways, preserving protein structure and function and attenuating AGE-mediated cellular injury in models of hyperglycemia and aging. Thus, targeting glycation represents a key strategy to protect cells from glycoxidative damage and associated pathological sequelae [54]. Compound **2** demonstrated a fourfold increase in antiglycation activity compared to compound **1**, supporting its superior antioxidant potential as indicated by the other antioxidant assays. These findings are consistent with earlier studies showing that butylated hydroxycinnamic acid derivatives exhibit greater inhibitory activity against lipid peroxidation and protein glycation than their corresponding benzoic acid derivatives [34,55].

### 2.6. Anti-Inflammatory Activity

The anti-inflammatory activity of the synthesized molecules was evaluated both in vitro (lipoxygenase and cyclooxygenase inhibition) and in vivo (carrageenan-induced rat paw edema inhibition). The results are shown in Table 5.

Lipoxygenases are enzymes involved in the metabolism of arachidonic acid. They operate through a shared mechanism that causes the peroxidation of arachidonic or linoleic acid using molecular oxygen. These enzymes play important roles in both normal physiological functions and pathological processes, as they contribute to the production of bioactive lipid mediators that regulate cellular signaling, inflammation, and oxidative stress. Consequently, lipoxygenases have been associated with the development and progression of various conditions, including inflammatory diseases, cancer, cardiovascular disorders, and neurodegenerative diseases [56,57,58]. Both compounds exerted moderate LOX inhibitory potential (up to 50% at 100 μΜ). Despite the higher antioxidant capacity of compound **2** (as estimated by ROS scavenging and metal-reducing assays), it demonstrated similar LOX inhibitory activity to compound **1**. Thus, it can be assumed that this inhibitory effect is not related to their ability to scavenge free radicals produced by LOX but instead results from their capacity to interact with the enzyme and prevent the substrate from binding to the active site. Moreover, no activity was observed when higher concentration of linoleic acid was used, pointing out that the synthesized molecules could act as weak competitive LOX inhibitors.

In addition, compounds **1** and **2** were tested for their COX inhibitory potential. Both of them exhibited more than double the inhibitory activity against COX-2 compared to COX-1. These results confirm earlier research projects, in which di-*tert*-butylphenol derivatives have been shown to act as dual LOX/COX2 inhibitors, providing a novel, safer therapeutic strategy for inflammatory conditions [59]. Furthermore, COX inhibitory activity of compounds **1** and **2** is similar to that of previous di-*tert*-butyl analogs designed and synthesized in our laboratory, strengthening the claim that such derivatives could act as selective COX2 inhibitors [55].

Finally, the compounds were evaluated in vivo for their carrageenan-induced paw edema inhibition. Carrageenan-induced inflammation initially triggers the release of histamine and serotonin approximately 90 min after administration, followed by the secretion of kinins around 2.5 h post-injection. In the later phase, occurring beyond 2.5 h, prostaglandins are generated and pro-inflammatory cytokines are released. The inflammatory reaction subsides about 5 h after carrageenan administration [60]. In our work, edema was estimated 3.5 h after the carrageenan administration. Compound **1** exerted similar activity to ibuprofen, while compound **2** was significantly more active against paw edema. COX-2 seems to play a key role in prostaglandin generation during the latter phase of carrageenan-induced inflammatory process; thus, the activity of the designed molecules could be, at least partly, attributed to their high inhibitory effect against COX-2. In addition, the anti-inflammatory capacity of the tested compounds is consistent with their antioxidant potential, pointing out that various oxidative processes could be part of the inflammatory response driven by carrageenan. Finally, the anti-inflammatory activity is not in accordance with the low LOX inhibitory activity of the compounds, indicating that LOX pathway may have only a minor contribution to the produced edema. All rats treated with the tested compounds showed no visible abnormalities, both on external examination and during autopsy, at the end of the experiment.

### 2.7. AchE Inhibition

**Figure 2:**
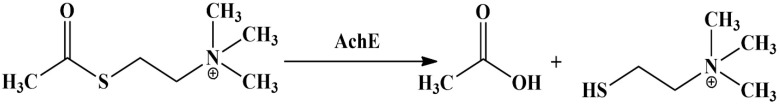
Acetylthiocholine Breakdown.

AchE is the primary pharmacological target of many anti-Alzheimer’s medications because the degeneration of cholinergic neurons is strongly associated with memory decline and cognitive dysfunction [61]. The inhibitory effects of the synthesized compounds on AchE were assessed by measuring their ability to prevent the hydrolysis of acetylthiocholine catalyzed by AchE obtained from rat brain homogenate (Figure 2).

Both compounds exerted moderate inhibitory activity with IC_50_ values of 312 μM and 368 μM, respectively. Their large molecular volume, due to the bulky *tert*-butyl substituents, may not allow them to reach the enzyme active site effectively. In previous research work, derivatives with less bulky groups, bearing proline and GABA moieties, demonstrated slightly lower IC_50_ values [62], pointing out that molecular volume may be a crucial factor for AchE inhibitory activity.

## 3. Materials and Methods

### 3.1. General

All commercially available reagents were obtained from Merck (Kenilworth, NJ, USA) or Sigma (St. Louis, MO, USA). Nuclear Magnetic Resonance (NMR) spectra (^1^H and ^13^C) were recorded using an AGILENT DD2-500 MHz spectrometer (Santa Clara, CA, USA). Melting points were determined with a MEL-TEMPII apparatus (Sigma-Aldrich, St. Louis, MO, USA). Elemental analyses (CHN) were conducted on a Perkin-Elmer 2400 elemental analyzer (Waltham, MA, USA). Reaction progress was monitored by thin-layer chromatography (TLC) using silica gel 60 F_25_ aluminum-backed plates (Merck), and spots were visualized under UV light. κ-Carrageenan and soybean lipoxygenase type I-B were sourced from Sigma (St. Louis, MO, USA). For in vivo experiments, adult Wistar rats (160–220 g, about 2 months old) were housed at the Centre of the School of Veterinary Medicine (EL-54-BIOexp-10), Aristotle University of Thessaloniki. The facility is registered with the official state veterinary authorities, and all procedures were conducted in accordance with European Directive 2010/63/EU. Experimental protocol received approval from the Animal Ethics Committee of the Prefecture of Central Macedonia (Approval No. 69787(294)).

### 3.2. Synthesis

Synthesis of compounds **1a** and **2a**

To a solution of the appropriate carboxylic acid (3 mmol, BHBA or BHCA) in dry dichloromethane, *1,1′-carbonyldiimidazole (CDI, 3.6 mmol)* was added, and the mixture was stirred at room temperature for 40 min. Afterwards, methyl pyrrolidine-2-carboxylate hydrochloride (L-proline methyl ester, 3.6 mmol) was introduced at the reaction mixture and stirred at room temperature for 24 h. After the completion of the reaction the mixture was washed sequentially with 5% HCl, 5% NaHCO_3_, and saturated NaCl solution. The organic phase was dried over anhydrous Na_2_SO_4_. Finally, the solvent was removed under reduced pressure, and the crude product was purified by flash column chromatography.

Synthesis of Compounds **1b** and **2b**

The respective proline methyl ester amide (**1a** or **2a**), obtained from the previous synthetic step, was dissolved in 1,4-dioxane (20 mL) and mixed with an equal volume of 5% aqueous NaOH solution. The reaction mixture was stirred for 45 min. Following this, dioxane was removed under reduced pressure, and the resulting aqueous layer was acidified with 10% HCl. The acidic solution was then extracted with ethyl acetate (3 × 20 mL). The combined organic layers were dried over anhydrous Na_2_SO_4_, and the solvent was subsequently removed under reduced pressure.

Synthesis of the final Compounds **1** and **2**

The corresponding proline amide (1 mmol) was dissolved in dry dichloromethane, with the addition of a small volume of DMF (up to 0.5 mL) to enhance solubility. Methyl 4-aminobutanoate hydrochloride (1.2 mmol) and DMAP (1.2 mmol) were added, followed, after 15 min, by DCC (1.2 mmol). The reaction mixture was stirred at room temperature overnight. Upon completion, the mixture was filtered, and the filtrate was sequentially washed with 5% HCl, 5% NaHCO_3_, and saturated NaCl solution. The organic phase was dried over anhydrous Na_2_SO_4_, and the solvent was removed under reduced pressure. The crude product was then purified by flash column chromatography.

Methyl 1-(3,5-di-*tert*-butyl-4-hydroxybenzoyl)pyrrolidine-2-carboxylate (**1a**):

Flash column chromatography (ethyl acetate/petroleum ether 1/2). Pale yellow solid, yield 75%, mp. 133–135 °C.

**IR (nujol)**: 3423 (O-H), 3221 (N-H), 1706 (C=O ester), 1631 (C=O amide), 1600, 1578 (C-C aromatic) cm^−1^.

**^1^H-NMR (CDCl_3_)**: 7.45 (s, 2H, aromatic), 5.45 (s, 1H, -OH), 4.65 (dd *J*: 7.9, 3.1 Hz, 1H, 2-pyrrolidine), 3.76 (s, 3H, -OCH_3_), 3.69–3.74 (m, 2H, 5-pyrrolidine), 2.28–2.32 (m, 1H, 3-pyrrolidine), 1.96–2.03 (m, 2H, 3-pyrrolidine, 4-pyrrolidine), 1.84–1.92 (m, 1H, 4-pyrrolidine), 1.42 (s, 18H, -C(CH_3_)_3_)**.**

**^13^C-NMR (CDCl3)**: 173.03 (1C, -**C**OOCH_3_), 170.23 (1C, -**C**O-N), 155.75 (1C, 4-aromatic), 135.37 (2C, 3,5-aromatic), 126.75 (1C, 1-aromatic), 125.06 (2C, 2,6-aromatic), 58.47 (1C, 2-pyrrolidine), 52.14 (1C, -O**C**H_3_), 50.14 (1C, 5-pyrrolidine), 34.37 (6C, (**C**H_3_)_3_C-), 30.16 (6C, (CH_3_)_3_**C**-), 29.32 (1C, 3-pyrrolidine), 25.65 (1C, 4-pyrrolidine).

1-(3,5-di-*tert*-butyl-4-hydroxybenzoyl)pyrrolidine-2-carboxylic acid (**1b**):

Pale yellow solid, yield 95%, mp. 158–161 °C.

**IR (nujol)**: 3405 (O-H), 3252 (COO-H), 3206 (N-H), 1731 (C=O carboxylic), 1630 (C=O amide), 1600, 1572 (C-C aromatic) cm^−1^.

**^1^H-NMR (CDCl_3_)**: 9.95 (s, 1H, -COOH), 7.41 (s, 2H, aromatic), 5.53 (s, 1H, -OH), 4.71 (dd *J*: 8.1, 3.3 Hz, 1H, 2-pyrrolidine), 3.66 (t *J*: 12.2 Hz, 2H, 5-pyrrolidine), 2.21–2.30 (m, 2H, 3-pyrrolidine), 1.83–2.00 (m, 2H, 4-pyrrolidine), 1.41 (s, 18H, (CH_3_)_3_C-).

**^13^C-NMR (CDCl_3_)**: 174.48 (1C, -**C**OOH), 171.28 (1C, -**C**O-N), 156.12 (1C, 4-aromatic), 135.63 (2C, 3,5-aromatic), 125.67 (1C, 1-aromatic), 125.05 (2C, 2,6-aromatic), 60.12 (1C, 2-pyrrolidine), 50.70 (1C, 5-pyrrolidine), 34.39 (6C, (**C**H_3_)_3_C-), 30.13 (6C, (CH_3_)_3_**C**-), 28.37 (1C, 3-pyrrolidine), 25.35 (1C, 4-pyrrolidine).

Methyl 4-(1-(3,5-di-*tert*-butyl-4-hydroxybenzoyl)pyrrolidine-2-carboxamido)butanoate (**1**):

Flash column chromatography (ethyl acetate). White powder, yield 60%, mp. 75–77 °C.

**IR (nujol)**: 3406 (O-H), 3215 (N-H), 1712 (C=O ester), 1632, 1627 (C=O amide), 1600, 1572 (C-C aromatic) cm^−1^.

**^1^H-NMR (CDCl_3_)**: 7.37 (s, 2H, aromatic), 5.48 (s, 1H, -OH), 4.74 (dd *J*: 8.0, 3.2 Hz, 1H, 2-pyrrolidine), 3.83 (s, 3H, -OCH_3_), 3.56–3.62 (m, 2H, 5-pyrrolidine), 3.22–3.27 (m, 2H, -NH-CH**_2_**), 2.34 (t *J*: 7.3 Hz, 2H, -CH**_2_**-C=O), 2.02–2.10 (m, 2H, 3-pyrrolidine), 1.80–1.91 (m, 4H, 4-pyrrolidine, -NH-CH2-CH**_2_**-), 1.43 (s, 18H, -C(CH_3_)_3_).

**^13^C-NMR (CDCl_3_)**: 173.63 (1C, -**C**OOCH_3_), 172.10, 171.35 (2C, -**C**O-N), 155.87 (1C, 4-aromatic), 135.61 (2C, 3,5-aromatic), 126.71 (1C, 1-aromatic), 124.90 (2C, 2,6-aromatic), 59.94 (1C, 2-pyrrolidine), 51.57 (1C, -O**C**H_3_), 50.87 (1C, 5-pyrrolidine), 38.84 (1C, -NH-**C**H_2_), 34.38 (6C, (**C**H_3_)_3_C-), 31.77 (1C, -**C**H_2_-COOCH_3_), 30.13 (6C, (CH_3_)_3_**C**-), 26.90 (1C, 3-pyrrolidine), 25.63 (1C, 4-pyrrolidine), 24.63 (1C, -C**-C**H_2_-C).

Anal. Calculated for C_25_H_38_N_2_O_5_: C, 67.24; H, 8.58, N, 6.27%. Found: C, 67.79; H, 8.36; N, 6.07%.

(*E*)-methyl 1-(3-(3,5-di-*tert*-butyl-4-hydroxyphenyl)acryloyl)pyrrolidine-2-carboxylate (**2a**):

Flash column chromatography (ethyl acetate/petroleum ether 1/2). Pale yellow solid, yield 73%, mp. 141–143 °C.

**IR (nujol)**: 3400 (O-H), 3196 (N-H), 1708 (C=O ester), 1642 (C=O amide), 1602, 1581 (C-C aromatic) cm^−1^.

**^1^H-NMR (CDCl_3_)**: 7.67 (d *J*: 15.9 Hz, 1H, Ph-C**H**=CH-), 7.34 (s, 2H, aromatic), 6.55 (d *J*: 15.9 Hz, 1H, Ph-CH=C**H**-), 5.45 (s, 1H, -OH), 4.61 (dd *J*: 8.1, 3.4 Hz, 1H, 2-pyrrolidine) 3.69–3.88 (m, 2H, 5-pyrrolidine), 3,74 (s, 3H, -OCH_3_), 2.09–2.36 (m, 4H, 3,4-pyrrolidine) 1.41 (s, 18H, -C(CH_3_)_3_).

**^13^C-NMR (CDCl3)**: 172.98 (1C, -**C**OOCH_3_), 165.40 (1C, -**C**O-N), 155.84 (1C, 4-aromatic), 144.05 (Ph-**C**H=CH-), 136.19 (2C, 3,5-aromatic), 126.47 (1C, 1-aromatic), 125.19 (2C, 2,6-aromatic), 114.65 (Ph-CH=**C**H-), 58.98 (1C, 2-pyrrolidine), 52.17 (1C, -O**C**H_3_), 46.99 (1C, 5-pyrrolidine), 34.28 (6C, (**C**H_3_)_3_C-), 30.15 (6C, (CH_3_)_3_**C**-), 29.18 (1C, 3-pyrrolidine), 24.85 (1C, 4-pyrrolidine).

(*E*)-1-(3-(3,5-di-*tert*-butyl-4-hydroxyphenyl)acryloyl)pyrrolidine-2-carboxylic acid (**2b**):

Pale yellow solid, yield 90%, mp. 175–177 °C.

**IR (nujol)**: 3405 (O-H), 3252 (COO-H), 3200 (N-H), 1732 (C=O carboxylic), 1631 (C=O amide), 1600, 1572 (C-C aromatic) cm^−1^.

**^1^H-NMR (CDCl_3_)**: 10.17 (s, 1H, -COOH), 7.64 (d *J*: 15.9 Hz, 1H, Ph-C**H**=CH-), 7.31 (s, 2H, aromatic), 6.57 (d *J*: 15.9 Hz, 1H, Ph-CH=C**H**-), 5.40 (s, 1H, -OH), 4.57 (dd *J*: 8.0, 3.2 Hz, 1H, 2-pyrrolidine) 3.72–3.88 (m, 2H, 5-pyrrolidine), 2.14–2.33 (m, 4H, 3,4-pyrrolidine) 1.41 (s, 18H, -C(CH_3_)_3_).

**^13^C-NMR (CDCl_3_)**: 172.61 (1C, -**C**OOH), 167,43 (1C, -**C**O-N), 155.08 (1C, 4-aromatic), 144.29 (Ph-**C**H=CH-), 137.21 (2C, 3,5-aromatic), 127.12 (1C, 1-aromatic), 126.03 (2C, 2,6-aromatic), 114.59 (Ph-CH=**C**H-), 59.76 (1C, 2-pyrrolidine), 48.17 (1C, 5-pyrrolidine), 34.17 (6C, (**C**H_3_)_3_C), 30.21 (6C, (CH_3_)_3_**C**-), 29.53 (1C, 3-pyrrolidine), 25.12 (1C, 4-pyrrolidine).

(*E*)-methyl 4-(1-(3-(3,5-di-*tert*-butyl-4-hydroxyphenyl)acryloyl)pyrrolidine-2-carboxamido)butanoate (**2**):

Flash column chromatography (ethyl acetate/petroleum ether 1/2). White solid, yield 68%, mp. 118–121 °C.

**IR (nujol)**: 3408 (O-H), 3215 (N-H), 1712 (C=O ester), 1632, 1627 (C=O amide), 1600, 1574 (C-C aromatic) cm^−1^

**^1^H-NMR (CDCl_3_)**: 7.69 (d *J*: 15.8 Hz, 1H, Ph-C**H**=CH-) 7.37 (s, 2H, aromatic), 6.56 (d *J*: 15.8 Hz, 1H, Ph-CH=C**H**-), 5.50 (s, 1H, -OH), 4.72 (dd *J*: 8.1, 3.2 Hz, 1H, 2-pyrrolidine), 3.65 (s, 3H, -OCH_3_), 3.60–3.64, 3.74–3.79 (m, 2H, 5-pyrrolidine), 3.20–3.33 (m, 2H, -NH-C**H_2_**-), 2.35 (t *J*: 7.1 Hz, 2H, -C**H_2_**-C=O), 2.10–2.17, 2.47–2.52 (m, 2H, 3-pyrrolidine), 1.79–1.91 (m, 4H, 4-pyrrolidine, -NH-CH_2_-C**H_2_**-), 1.46 (s, 18H, -C(CH_3_)_3_).

**^13^C-NMR (CDCl_3_)**: 173.62 (1C, -**C**OOCH_3_), 171.35, 166.93 (2C, -**C**O-N), 155.89 (1C, 4-aromatic), 144.52 (Ph-**C**H=CH-), 136.31 (2C, 3,5-aromatic), 126.20 (1C, 1-aromatic), 125.30 (2C, 2,6-aromatic), 114.33 (Ph-CH=**C**H-), 59.93 (1C, 2-pyrrolidine), 51.58 (1C, -O**C**H_3_), 47.45 (1C, 5-pyrrolidine), 38.72 (1C, -NH-**C**H_2_), 34.39 (6C, (**C**H_3_)_3_C-), 31.37 (1C, -**C**H_2_-COOCH_3_), 30.14 (6C, (CH_3_)_3_**C**-), 26.86 (1C, 3-pyrrolidine), 25.01 (1C, 4-pyrrolidine), 24.76 (1C, -C-**C**H_2_-C).

Anal. Calculated for C_27_H_40_N_2_O_5_: C, 68.62; H, 8.55, N, 5.93%. Found: C, 68.55; H, 8.09; N, 6.12%.

### 3.3. Biological Evaluation

The in vitro assays were performed by recording the absorption of the resulting final mixtures at various times and concentrations according to every experiment, using UV-VIS spectrophotometer (Shimanzu UV-PharmaSpac 1700, Kyoto, Japan) or fluorometer and poly(methyl methacrylate) (PMMA) cuvettes with 10 mm width. The absorption peaks that the antioxidant assays were executed with ranged between 240 and 734 nm, at which some of the compounds may absorb. Thus, solutions containing the compounds alone were used as blank and their absorption was subtracted from the final recorded absorbance. All the tests were performed three times, and the mean values of the measurements are reported. The standard deviation from the mean was always ±10%.

#### 3.3.1. Radical Scavenging Assays

##### In Vitro Interaction with the Stable Free Radical 1,1-Diphenyl-2-Picrylhydrazyl (DPPH)

The DPPH radical scavenging assay evaluates antioxidant activity through hydrogen atom transfer from an antioxidant to the stable DPPH radical, resulting in a color change from violet to yellow. A 4 mM DPPH solution is prepared in ethanol and diluted to 0.4 mM. Tested compounds are dissolved in ethanol to obtain corresponding stock solutions, which are further diluted to the desired concentrations. Equal volumes of DPPH and sample solutions are mixed and incubated for 30 min, after which absorbance is measured at 517 nm. Ethanol serves as the blank. Radical scavenging activity is calculated as percentage inhibition using the control and sample absorbance values [63].

##### ABTS·^+^ Radical Scavenging

The ABTS assay measures antioxidant activity through decolorization of the ABTS radical cation in the presence of hydrogen-donating compounds. The ABTS·^+^ radical is generated by reacting ABTS with potassium persulfate and allowing the mixture to stand in the dark for 16 h. Prior to use, the radical solution is diluted with ethanol to obtain an absorbance of about 0.700. The assay is performed by mixing diluted ABTS·^+^ solution with sample solutions at various concentrations, followed by 30 min incubation. Absorbance is measured at 734 nm, and radical scavenging activity is expressed as percentage inhibition relative to the control [64].

##### Hydrogen Peroxide Scavenging Potential

Hydrogen peroxide scavenging activity was evaluated using a standard method. A hydrogen peroxide solution (40 mM) was prepared in potassium phosphate buffer (pH 7.4), while stock solutions of the tested compounds were prepared in ethanol and diluted to the required concentrations. The assay was performed by mixing sample solution with hydrogen peroxide and phosphate buffer, followed by incubation in the dark at 37 °C for 15 min. Absorbance was measured at 230 nm against appropriate blanks. Ascorbic acid was used as reference antioxidant. Scavenging activity was expressed as percentage inhibition based on the difference between control and sample absorbance values [65].

#### 3.3.2. Metal Cations Reducing Activity

##### Ferric-Reducing Power Assay (RP)

Ferric-reducing antioxidant power was assessed by evaluating the ability of the compounds to reduce ferric ions derived from potassium ferricyanide, leading to Prussian blue formation upon reaction with FeCl_3_. Solutions of potassium ferricyanide, trichloroacetic acid, and ferric chloride were prepared, along with stock solutions of the tested compounds in dimethylsulfoxide (DMSO). The assay mixture containing sample, phosphate buffer (pH 6.6), and potassium ferricyanide was incubated at 50 °C, followed by addition of trichloroacetic acid. After further processing with FeCl_3_, absorbance was measured at 700 nm against a blank. Reducing activity was expressed relative to a reference antioxidant (Trolox) as percentage of ferric-reducing power [66].

##### Ferric-Reducing Antioxidant Potential (FRAP)

Ferric-reducing antioxidant power (FRAP) was evaluated based on the reduction in ferric to ferrous ions, followed by formation of a blue ferrous–TPTZ (2,4,6-tri-2-pyridinyl-1,3,5-triazine) complex. Acetate buffer, TPTZ, and FeCl_3_ solutions were prepared and combined to form the FRAP reagent. The assay was performed by mixing FRAP reagent with diluted sample solutions and incubating for 8 min, after which absorbance was measured at 593 nm against a blank. Sample stock solutions were prepared in ethanol and diluted to the desired concentrations. Antioxidant capacity was assessed relative to trolox and expressed as percentage ferric-reducing power compared to the reference [66].

##### Total Antioxidant Capacity (TAC) via the Phosphomolybdate Assay

Total antioxidant capacity was assessed through the reduction of molybdenum (VI) to molybdenum (V), resulting in formation of a green phosphomolybdenum (Mo^5+^) complex. The TAC reagent, containing sulfuric acid, sodium phosphate, and ammonium molybdate, was prepared and mixed with sample solutions at various concentrations. The reaction mixtures were incubated at elevated temperature, after which absorbance was measured at 695 nm against a blank. Antioxidant activity was evaluated relative to Trolox and expressed as percentage molybdenum reducing power [63].

#### 3.3.3. Iron-Binding Ability

Ferrous ion chelating activity of phenolic compounds was evaluated based on their ability to bind Fe^2+^ ions and inhibit formation of the Fe^2+^–ferrozine complex. Solutions of FeSO_4_, ferrozine, and sample compounds were prepared, and the assay was performed by mixing Fe^2+^ with the sample followed by addition of ferrozine. After incubation at room temperature, absorbance was measured at 562 nm. EDTA was used as a reference chelating agent. Chelating activity was expressed as percentage inhibition of the Fe^2+^–ferrozine complex formation relative to the control [67].

#### 3.3.4. Cellular Components Protective Potential

##### In Vitro Lipid Peroxidation Inhibition

The rat liver microsomal fraction was inactivated with heat at 90 °C for 90 s and ascorbic acid (0.2 mM) and Fe_2_SO_4_ (10 μΜ) were added for the induction of its peroxidation. The dissolved in DMSO compounds, were added (concentrations 1 μΜ–1 mM). Aliquots were removed from the reaction mixture for 45 min (37 °C). The induction of peroxidation was assessed at 535/600 nm as 2-thiobarbituric acid reactive material. All solvents and compounds did not affect the assay [55].

##### In Vitro Protein Glycation Inhibition

Bovine Serum Albumin (BSA, 4 mg/mL) was incubated with fructose (250 mM), Cu^2+^ (10 μM), and NaN_3_ (0.015%) in 120 mM phosphate buffer (pH 7.4) at 37 °C for 72 h. The glycation product was purified by protein precipitation and subsequent washing with trichloroacetic acid (TCA). The concentration of the glycation-modified protein was determined using fluorescence measurements (excitation at 340 nm, emission at 410 nm). Control incubations, conducted under identical conditions but without fructose, were used for comparison. All fluorescence values were referenced to a standard quinine sulphate solution (1 μg/mL) [55]. None of the compounds showed any degradation during the experiment.

#### 3.3.5. Anti-Inflammatory Activity

##### In Vitro Assessment of Cyclooxygenase-1 and -2 Activity

The influence of selected compounds on the enzymatic activity of COX-1 and COX-2 was assessed using ovine COX-1 and human recombinant COX-2 enzymes, as provided in the “COX Inhibitor Screening Assay” kit (Cayman Chemical Co., Ann Arbor, MI, USA). This assay quantifies prostaglandin F2α (PGF2α) generated through the SnCl_2_-mediated reduction in prostaglandin H_2_ (PGH_2_), a product of COX activity, using an enzyme immunoassay with an antibody that recognizes a broad range of prostaglandins. The inhibition of enzyme activity by the test compounds was evaluated in the presence of arachidonic acid at a concentration of 0.1 μM. Test compounds were added to the reaction mixture at a final concentration of 50 μM. The enzyme concentration for both COX isoforms was maintained at 10^−9^ IU/mL [68].

##### In Vitro Evaluation of Lipoxygenase Activity

Lipoxygenase inhibitory activity was evaluated using soybean lipoxygenase in Tris/HCl buffer (pH 9.0). The reaction mixture contained buffer, enzyme solution, and test compounds prepared in ethanol, and the reaction was initiated by addition of sodium linoleate as substrate. Absorbance was monitored at 234 nm at regular intervals for 7 min at 28 °C. Nordihydroguaiaretic acid (NDGA) was used as the reference inhibitor. Inhibition type was assessed using substrate concentrations exceeding the saturation level [69].

##### In Vivo Evaluation of Anti-Inflammatory Activity

An aqueous carrageenan solution (1% *w*/*v*) and solutions of the compounds (0.15 mmol/kg, with a few drops of Tween 80) were prepared. Each experimental group of 6 rats received an intraperitoneal injection of the compound at a dose of 0.15 mmol/kg. After 5 min, 0.1 mL of carrageenan was administered intradermally into the hind paw of both treated and control groups. After 3.5 h, the weight difference between the hind paws was measured and expressed as a percentage increase relative to the carrageenan control group [55].

#### 3.3.6. In Vitro Evaluation of Acetylcholinesterase Activity

The test compounds, prepared in 60% ethanol, were incubated with brain homogenate from untreated rats (prepared in 0.1 M phosphate buffer, pH 8) along with 0.5 mM acetylthiocholine. Enzymatic activity was determined by measuring the formation of the yellow-colored product resulting from the reaction between liberated thiocholine and 5,5′-dithiobis-(2-nitrobenzoic acid) (DTNB) at an absorbance of 412 nm. The solvent system was evaluated prior to the assay and was confirmed to have no impact on the measurement [62].

## 4. Conclusions

In the present study, two novel hybrid molecules incorporating butylated phenolic moieties, proline, and GABA were successfully designed, synthesized, and biologically evaluated as multifunctional agents targeting oxidative stress and inflammation. The biological evaluation demonstrated that both compounds possess significant antioxidant and anti-inflammatory properties, with compound **2** consistently exhibiting superior activity across most experimental models. Specifically, compound **2** showed strong radical scavenging capacity against ABTS·^+^ and hydrogen peroxide, enhanced ferric-reducing ability, and notable iron-chelating activity, indicating a robust redox-modulating potential. Moreover, its pronounced inhibitory effect on lipid peroxidation and protein glycation highlights its cytoprotective capacity under oxidative and glycoxidative stress conditions. Both compounds demonstrated selective inhibition of COX-2 over COX-1, combined with moderate lipoxygenase inhibition and significant in vivo anti-inflammatory activity in the carrageenan-induced paw edema model. These findings suggest that the anti-inflammatory effects are primarily mediated through COX-2 inhibition and are further supported by their antioxidant properties. Additionally, moderate acetylcholinesterase inhibitory activity indicates potential relevance in neuroprotective applications, although further optimization may improve enzyme-target interactions. Overall, the results confirm that structural hybridization of butylated phenolic acids with proline and GABA yields multifunctional compounds with combined antioxidant, anti-inflammatory, and antiglycation properties. Among them, compound **2** emerges as a particularly promising lead candidate. These findings support the potential of this hybrid design strategy for the development of novel therapeutic agents targeting oxidative stress- and inflammation-related disorders, including neurodegenerative, cardiovascular, and metabolic diseases.

## 5. Limitations-Future Perspectives

This study presents certain limitations that should be considered. The biological evaluation was restricted to a limited number of compounds, which constrains the breadth of structure-activity relationship insights. In addition, most findings are based on in vitro assays and a single in vivo acute inflammation model, which may not fully reflect complex chronic disease conditions. Mechanistic investigations at the molecular level remain limited, and key pharmacokinetic parameters, including ADME properties and blood–brain barrier permeability, were not assessed.

Future work should focus on expanding the chemical library to better define structure-activity relationships and optimize biological activity. Further in vivo studies in disease-relevant models are required to validate therapeutic potential. Detailed mechanistic studies, alongside pharmacokinetic and toxicity evaluations, will be essential to support the development of these compounds as multi-target therapeutic agents.

## Figures and Tables

**Table 1:** IC_50_ values (μΜ) for compounds **1** and **2**, as well as reference compounds against DPPH, ABTS·^+^ and H_2_O_2_.

Compounds	DPPH	ABTS·^+^	H_2_O_2_
**1**	145	39	255
**2**	86	25	104
**Trolox**	58	13.5	-
**Ascorbic acid**	-	-	113

DPPH and ABTS·^+^: 30 min incubation. -: not tested. Trolox: 6-hydroxy-2,5,7,8-tetramethylchroman-2-carboxylic acid. All the tests were performed three times, and the mean values of the measurements are reported. The standard deviation from the mean was always within ±10%.

**Table 2:** Activity of various concentrations of the synthesized compounds in FRAP, RP, and TAC assay, expressed as percent (%) activity of trolox.

Compounds	FRAP Assay				RP Assay				TAC Assay			
Concentrations	200 μΜ	100 μΜ	50 μΜ	25 μΜ	500 μΜ	200 μΜ	100 μΜ	50 μΜ	500 μΜ	200 μΜ	100 μΜ	50 μΜ
**1**	7.0	6.5	5.7	6.3	9.6	8.4	5.9	5.4	8.5	7.3	7.6	5.6
**2**	91.1	72.2	47.9	35.8	29.7	26.4	20.2	18.4	68.9	71.0	70.9	61.4

All the tests were performed three times, and the mean values of the measurements are reported. The standard deviation from the mean was always within ±10%.

**Table 3:** Fe^2+^ binding activity of the designed compounds and EDTA.

Compounds	% Binding Activity
**1**	32.4
**2**	61.3
**EDTA**	91.6

All the tests were performed three times, and the mean values of the measurements are reported. The standard deviation from the mean was always within ±10%.

**Table 4:** Inhibitory activity of the designed molecules against lipid peroxidation and oxidative protein glycation. Activity of reference compounds is depicted as well.

Compounds	Lipid Peroxidation-IC_50_ (μM)	Protein Glycation-% Inhibition at 1 mM
**1**	418	10
**2**	17.5	44
**Trolox**	25	-
**Aminoguanidine**	-	56

-: not tested. All the tests were performed three times, and the mean values of the measurements are reported. The standard deviation from the mean was always within ±10%.

**Table 5:** Anti-inflammatory activity of the designed compounds.

Compounds	LOX (% Inhibition at 100 μΜ)	COX-1 (% Inhibition at 50 μΜ)	COX-2 (% Inhibition at 50 μΜ)	% Rat Paw Edema Inhibition
**1**	47	42	87	35 *
**2**	50	37	93	53 **
**Ibuprofen**	-	68	46	36 *
**NDGA**	94	-	-	-

-: not tested. NDGA: Nor-dihydroguaiaretic acid. All the in vitro tests were performed three times, and the mean values of the measurements are reported. The standard deviation from the mean was always within ±10% **: *p* < 0.001, *: *p* < 0.01 (as calculated via the GraphPad Student’s *t*-test).

## Data Availability

The original contributions presented in this study are included in the article. Further inquiries can be directed to the corresponding author.
